# Supplementary material for: Low Dose Low-Molecular-Weight Heparin for Thrombosis Prophylaxis: Systematic Review with Meta-Analysis and Trial Sequential Analysis
Source: J Clin Med. 2019 Nov 21;8(12):2039. doi: 10.3390/jcm8122039 (PMC6947554; doi:10.3390/jcm8122039)
Supplement: Supplementary file 1 [file jcm-08-02039-s001.pdf]

Table of contents

PRISMA checklist 2

Tables

Supplementary Table S1. Search strategy 4

Supplementary Table S2. Characteristics of included randomized trials, stratified by patient type . 6

Supplementary Table S3. Ongoing trials........ 10

Supplementary Table S4. Risk of bias assessment........ **Error! Bookmark not defined.**

Supplementary Table S5. Sensitivity analysis: best-worse and worst-best case scenario's........ 11

Supplementary Table S6. Sensitivity analysis: trials with publication year ≥ 2005 16

Supplementary Table S7. Sensitivity analysis: including LMWH types not a priori defined ..... **Error! Bookmark not defined.**

Supplementary Table S8. Subgroup analysis: co-primary outcomes........ 16

Supplementary Table S9. Sensitivity analysis: proportion of SAE and cumulative SAE 13

Figures

Supplementary Figure S1. Forest plot of SAE, stratified for patient type 17

Supplementary Figure S2. Forest plot of non-major bleeding, stratified for patient type 18

Supplementary Figure S3. Forest plot of any VTE, stratified for patient type........ 19

Supplementary Figure S4. Funnel plot of all-cause mortality 20

Supplementary Figure S5. Funnel plot of symptomatic VTE 20

Supplementary Figure S6. Funnel plot of major bleeding........ 21

Supplementary Figure S7. Funnel plot of any VTE 21

References 22

PRISMA checklist.

Section/topic	#	Checklist item	Reported on page #
**TITLE**			
Title	1	Identify the report as a systematic review, meta-analysis, or both.	1
**ABSTRACT**			
Structured summary	2	Provide a structured summary including, as applicable: background; objectives; data sources; study eligibility criteria, participants, and interventions; study appraisal and synthesis methods; results; limitations; conclusions and implications of key findings; systematic review registration number.	1
**INTRODUCTION**			
Rationale	3	Describe the rationale for the review in the context of what is already known.	1
Objectives	4	Provide an explicit statement of questions being addressed with reference to participants, interventions, comparisons, outcomes, and study design (PICOS).	1
**METHODS**			
Protocol and registration	5	Indicate if a review protocol exists, if and where it can be accessed (e.g., Web address), and, if available, provide registration information including registration number.	2
Eligibility criteria	6	Specify study characteristics (e.g., PICOS, length of follow-up) and report characteristics (e.g., years considered, language, publication status) used as criteria for eligibility, giving rationale.	2
Information sources	7	Describe all information sources (e.g., databases with dates of coverage, contact with study authors to identify additional studies) in the search and date last searched.	2
Search	8	Present full electronic search strategy for at least one database, including any limits used, such that it could be repeated.	Suppl. Table 1
Study selection	9	State the process for selecting studies (i.e., screening, eligibility, included in systematic review, and, if applicable, included in the meta-analysis).	2
Data collection process	10	Describe method of data extraction from reports (e.g., piloted forms, independently, in duplicate) and any processes for obtaining and confirming data from investigators.	3
Data items	11	List and define all variables for which data were sought (e.g., PICOS, funding sources) and any assumptions and simplifications made.	3
Risk of bias in individual studies	12	Describe methods used for assessing risk of bias of individual studies (including specification of whether this was done at the study or outcome level), and how this information is to be used in any data synthesis.	3
Summary measures	13	State the principal summary measures (e.g., risk ratio, difference in means).	3
Synthesis of results	14	Describe the methods of handling data and combining results of studies, if done, including measures of consistency (e.g., I^2) for each meta-analysis.	4

Section/topic	#	Checklist item	Reported on page #
Risk of bias across studies	15	Specify any assessment of risk of bias that may affect the cumulative evidence (e.g., publication bias, selective reporting within studies).	3
Additional analyses	16	Describe methods of additional analyses (e.g., sensitivity or subgroup analyses, meta-regression), if done, indicating which were pre-specified.	4
**RESULTS**			
Study selection	17	Give numbers of studies screened, assessed for eligibility, and included in the review, with reasons for exclusions at each stage, ideally with a flow diagram.	5; Fig 2
Study characteristics	18	For each study, present characteristics for which data were extracted (e.g., study size, PICOS, follow-up period) and provide the citations.	Suppl. Table 2
Risk of bias within studies	19	Present data on risk of bias of each study and, if available, any outcome level assessment (see item 12).	Suppl. Table 4
Results of individual studies	20	For all outcomes considered (benefits or harms), present, for each study: (a) simple summary data for each intervention group (b) effect estimates and confidence intervals, ideally with a forest plot.	5-11
Synthesis of results	21	Present results of each meta-analysis done, including confidence intervals and measures of consistency.	5-11
Risk of bias across studies	22	Present results of any assessment of risk of bias across studies (see Item 15).	5-11, Suppl. Figs 4-7
Additional analysis	23	Give results of additional analyses, if done (e.g., sensitivity or subgroup analyses, meta-regression [see Item 16]).	Suppl. Table 5-9
**DISCUSSION**			
Summary of evidence	24	Summarize the main findings including the strength of evidence for each main outcome; consider their relevance to key groups (e.g., healthcare providers, users, and policy makers).	12
Limitations	25	Discuss limitations at study and outcome level (e.g., risk of bias), and at review-level (e.g., incomplete retrieval of identified research, reporting bias).	13
Conclusions	26	Provide a general interpretation of the results in the context of other evidence, and implications for future research.	14
**FUNDING**			
Funding	27	Describe sources of funding for the systematic review and other support (e.g., supply of data); role of funders for the systematic review.	14

Table S1. Search strategy.

**PUBMED/MEDLINE**
("Heparin, Low-Molecular-Weight"[Mesh] OR low-molecular-weight heparin*[tiab] OR lmwh [tiab] OR "Nadroparin"[Mesh] OR nadroparin*[tiab] OR fraxiparin*[tiab] OR cy 216[tiab] OR cy216[tiab] OR lmf cy216[tiab] OR seleparin*[tiab] OR "Enoxaparin"[Mesh] OR enoxaparin*[tiab] OR clexane[tiab] OR klexane[tiab] OR lovenox[tiab] OR emt 966[tiab] OR emt 967[tiab] OR pk-10,169[tiab] OR pk 10169[tiab] OR pk10169[tiab] OR "Dalteparin"[Mesh] OR dalteparin*[tiab] OR fragmin*[tiab] OR tedelparin[tiab] OR kabi 2165[tiab] OR fr 860[tiab] OR "tinzaparin"[Supplementary Concept] OR tinzaparin*[tiab] OR innohep[tiab] OR logiparin[tiab] OR lhn-1[tiab] OR ardeparin*[tiab] OR normiflo[tiab] OR "bemiparin"[Supplementary Concept] OR bemiparin*[tiab] OR zibor*[tiab] OR "certoparin"[Supplementary Concept] OR certoparin*[tiab] OR mono-embolex[tiab] OR embolex[tiab] OR sandoparin[tiab] OR cy222 OR cy 222 OR "reviparin"[Supplementary Concept] OR reviparin*[tiab] OR clivarin[tiab] OR lu 47311[tiab] OR "parnaparin"[Supplementary Concept] OR parnaparin[tiab] OR cb-01-05-mmx[tiab] OR fluxum[tiab] OR "semuloparin"[Supplementary Concept] OR semuloparin*[tiab] OR ave5026[tiab])
AND
("Mortality"[Mesh:noexp] OR mortality[tiab] OR "Survival"[Mesh] OR survival [tiab] OR "Thrombosis"[Mesh] OR thrombos*[tiab] OR thrombot*[tiab] OR "Thromboembolism"[Mesh] OR thromboembol*[tiab] OR "Pulmonary Embolism"[Mesh] OR VTE[tiab] OR DVT[tiab])
AND
(randomized controlled trial[pt] OR randomi* [tiab] OR randomly[tiab] OR placebo[tiab] OR trial[ti] NOT (animals[mh] NOT humans[mh]))
**EMBASE**
('low molecular weight heparin'/exp OR ('low molecular' NEXT/3 heparin*):ab,ti OR lmwh:ab,ti OR (nadroparin* OR fraxiparin* OR cy216 OR 'cy 216' OR 'lmf cy216' OR seleparin* OR tedegliparin* OR enoxaparin OR clexane OR klexane OR lovenox OR emt966 OR 'emt 966' OR 'emt 967' OR 'pk 10,169' OR 'pk 10169' OR pk10169 OR dalteparin* OR fragmin* OR tedelparin OR 'kabi2165' OR 'kabi 2165' OR 'fr 860' OR fr860 OR tinzaparin* OR innohep OR logiparin OR 'lhn-1' OR ardeparin OR normiflo OR bemiparin OR zibor OR certoparin OR alphaparin OR 'mono-embolex' OR embolex OR sandoparin OR 'cy 222' OR cy222 OR 'reviparin sodium' OR clivarin* OR 'lu 47311' OR parnaparin OR 'cb-01-05-mmx' OR fluxum OR semuloparin OR ave5026):ab,ti)
AND
('mortality'/de OR 'mortality':ab,ti OR 'survival'/de OR survival:ab,ti OR 'thromboembolism'/exp OR thrombos*:ab,ti OR thrombot*:ab,ti OR thromboembol*:ab,ti OR 'pulmonary embolism' OR dvt:ab,ti OR vte:ab,ti)
AND
('randomized controlled trial'/exp OR (randomi*ed NEXT/3 controlled NEXT/3 (trial OR study)):ab,ti NOT ('animal'/exp OR 'nonhuman'/exp NOT 'human'/exp))
**COCHRANE CENTRAL**
(Low-molecular-weight heparin* OR lmwh OR nadroparin* OR fraxiparin* OR cy216 OR ''cy 216'' OR ''lmf cy216'' OR seleparin* OR tedegliparin* OR enoxaparin* OR clexane OR klexane OR lovenox OR emt966 OR ''emt 966'' OR ''emt 967'' OR ''pk-10,169'' OR ''pk 10169'' OR pk10169 OR dalteparin* OR fragmin* OR tedelparin OR kabi2165 OR ''kabi 2165'' OR ''fr 860'' OR fr860 OR tinzaparin* OR innohep OR logiparin OR ''lhn-1'' OR ardeparin OR normiflo OR bemiparin OR zibor OR certoparin OR alphaparin OR ''mono-embolex'' OR embolex OR sandoparin OR cy222 OR ''cy 222'' OR reviparin* OR clivarin OR ''lu 47311'' OR parnaparin OR ''cb-01-05-mmx'' OR fluxum OR semuloparin OR ave5026)
AND
(Mortality OR survival OR thrombos* OR thromboembol* OR thrombot* OR pulmonary embolism OR VTE OR DVT OR PE)
SEARCH IN: TRIALS

WEB OF SCIENCE
**TS=**(Low-molecular-weight heparin* OR lmwh OR nadroparin* OR fraxiparin* OR cy216 OR "cy 216" OR "lmf cy216" OR seleparin* OR tedegliparin* OR enoxaparin* OR clexane OR klexane OR lovenox OR emt966 OR "emt 966" OR "emt 967" OR "pk-10,169" OR "pk 10169" OR pk10169 OR dalteparin* OR fragmin* OR tedelparin OR kabi2165 OR"kabi 2165" OR fr 860 OR fr860 OR tinzaparin* OR innohep OR logiparin OR "lhn-1" OR ardeparin OR normiflo OR bemiparin OR zibor OR certoparin OR alphaparin OR "mono-embolex" OR embolex OR sandoparin OR cy222 OR "cy 222" OR reviparin* OR clivarin OR "lu-47311" OR parnaparin OR "cb-01-05-mmx" OR fluxum OR semuloparin OR ave5026)
AND
**TS =** (Mortality OR survival OR thrombos* OR thromboembol* OR thrombot* OR pulmonary embolism OR VTE OR DVT OR PE)
AND
(**TS =** (random* NEXT/3 trial*) OR **TS =** (random* NEXT/3 stud*) OR **TI=**trial* OR **TS =** (”randomized controlled" OR “randomised controlled”))

Table S2. Characteristics of included randomized trials, stratified by patient type.

Trial	Patient type	Sample size	Intervention	Duration of intervention	Control	Outcomes	Follow-up
**Acutely ill medical patients**							
Fraisse, 2000[1]	Critically ill	223	Nadroparin weight based: 44% 5700 IU, 56% 3800 IU, 1x daily	Mean 11.4 +- 6.0 days, max 21 days	Placebo	Mortality; SAE, any VTE; major bleeding	21 days
Mahe, 2005[2]	Acutely ill medical	2,474	Nadroparin 2850 IU, 1x daily	21 days or until end of hospitalisation	Placebo	Mortality; symptomatic VTE; major bleeding	21 days
Ozcan, 2004[3]	Allogeneic stem cell transplantation	38	Dalteparin 2500 IU, 1x daily	Day -9 to 100 post transplant	No intervention	Symptomatic VTE	9 months
Samama, 1999[4]	Acutely ill medical	735 (1102 total)	Enoxaparin 20mg, 1x daily	7 days	Placebo	Mortality; symptomatic VTE; major bleeding; any VTE	83-110 days
**Surgery**							
Baca, 1997[5]	Elective laparoscopic surgery (appendectomy or cholecystectomy)	718	Reviparin 1750 IU, 1x daily	Start at surgery, for at least 6 days	No intervention	Symptomatic VTE; major bleeding	7 days
Balas, 1992[6]	General surgery	189	Nadroparin 2850 IU, 1x daily	Start before surgery, at least 5-8 days	Placebo	Symptomatic VTE; major bleeding; any VTE	NS
Bergqvist, 1996[7]	Acute abdominal surgery	80	Tinzaparin 3500 IU, 1x daily	Start after surgery, duration unknown	Placebo	Mortality; major bleeding; any VTE	30 days
Burrows, 2001[8]	Elective or emergency caesarean section	76	Dalteparin 2500 IU, 1x daily	Start after surgery, for 5 days	Placebo	Mortality; symptomatic VTE; major bleeding	6 weeks
Dong, 2018[9]	Thoracic cancer surgery	111	Nadroparin, 2850 IU, 1x daily	Start 24h after surgery, for 7 days	No intervention	Major bleeding; any VTE	7 days
Marassi, 1993[10]	Abdominal cancer surgery	64	Nadroparin 3800 IU, 1x daily	Start 12h after surgery, for 7 days	No intervention	Any VTE	7 days
Melon, 1991[11]	Neurosurgery	130	Enoxaparin 20mg, 1x daily	Start after surgery, for 10 days	Placebo	Major bleeding; any VTE	10 days
Murugesan, 2010[12]	Major abdominal surgery	65	Nadroparin 3075 IU, 1x daily	Start 2h before surgery, for 7-9 days	No intervention	Symptomatic VTE; any VTE	10 days

Trial	Patient type	Sample size	Intervention	Duration of intervention	Control	Outcomes	Follow-up
Norberto Garcia, 2013[13]	Varicose vein surgery	264	Bemiparin 2500 IU, 1x daily	Start 6h after surgery, for 10 days	No intervention	Mortality; symptomatic VTE; major bleeding; any VTE	6 months
Nurmohamed, 1996[14]	Neurosurgery	485	Nadroparin, 2850 IU, 1x daily	Start 18-24h after surgery, for 10 days	Placebo	Mortality; symptomatic VTE; major bleeding; any VTE	56 days
Ockelford, 1989[15]	Major abdominal surgery	197	Dalteparin, 2500 IU, 1x daily	Start 1-2 h before surgery, for 10 days	Placebo	Mortality; major bleeding; clinically relevant non-major bleeding; any VTE	6 weeks
Pezzuoli, 1990[16]	General surgery	4,498	Nadroparin, 2850 IU, 1x daily	Start 2h before surgery, for 7 days	Placebo	Mortality; symptomatic VTE	10 days
Valle, 1988[17]	Major abdominal surgery	100	Parnaparin, 3200 IU, 1x daily	Start 2h before surgery, for 7 days	Placebo	Major bleeding; any VTE	NS
**Orthopedics or immobilisation**							
Van Adrichem, 2017 (POT-KAST)[18]	Knee arthroscopy	1543	Nadroparin 2850 IU, dalteparin 2500 IU, 1x daily (>100kg double dose)	8 days, start after arthroscopy	No intervention	Mortality, SAE, symptomatic VTE, major bleeding, clinically relevant non-major bleeding	3 months
Van Adrichem, 2017 (POT-CAST)[18]	Lower leg casting	1519	Nadroparin 2850 IU, dalteparin 2500 IU, 1x daily (>100kg double dose)	During immobilisation period	No intervention	Mortality, SAE, symptomatic VTE, major bleeding, clinically relevant non-major bleeding	3 months
Bruntink, 2017[19]	Below-the-knee fractures (ankle or foot) requiring plaster cast for >4 weeks	310	Nadroparin, 2850 IU, 1x daily	During immobilisation period	No intervention	Mortality; symptomatic VTE; major bleeding; any VTE	40 days
Fuji, 2008a[20]	Elective primary total hip arthroplasty patients	209 (436 total)	Enoxaparin 20mg, 1x daily	14 days	Placebo	Major bleeding; SAE; any VTE	90 days
Fuji, 2008b[20]	Elective primary total knee arthroplasty patients	189 (396 total)	Enoxaparin 20mg, 1x daily	14 days	Placebo	Major bleeding; SAE; any VTE	90 days
Jorgensen, 2002[21]	Plaster cast for at least 3 weeks, regardless of diagnosis	300	Tinzaparin, 3500 IU, 1x daily	During cast period	No intervention	Mortality; symptomatic VTE; major bleeding; SAE; any VTE	NS

Trial	Patient type	Sample size	Intervention	Duration of intervention	Control	Outcomes	Follow-up
Kock, 1995[22]	Plaster cast immobilisation, for several reasons including surgery	391	Certoparin, 3000 IU, 1x daily	During cast period	No intervention	Major bleeding; SAE; any VTE	15 days
Kujath, 1993[23]	Plaster cast for at least 7 days, for lower limb injury	253	Nadroparin, 2850 IU, 1x daily	During cast period	No intervention	Any VTE	NS
Lassen, 1991[24]	Elective hip replacement surgery	210	Tinzaparin, 50 IU/kg (median 3560 IU), 1x daily	Start 2h before surgery, for 7 days	Placebo	Mortality; symptomatic VTE; clinically relevant non-major bleeding; any VTE	10 days
Lassen, 2002[25]	Plaster cast immobilisation for at least 5 weeks, for lower leg fracture or achilles tendon rupture (with or without OK	440	Reviparin, 1750 IU, 1x daily	During cast period	Placebo	Mortality; symptomatic VTE; major bleeding; any VTE	40-50 days
Levine, 1996[26]	Elective knee surgery	246	Ardeparin, 50 IU/kg, 2x daily	14 days or until discharge	Placebo	Mortality; major bleeding; any VTE	14 days
Xiao-Li, 2001[27]	Hip and knee surgery	46	Nadroparin, 41-62 IU / kg, 1x daily	10 days	NS	Major bleeding; any VTE	NS
Sorensen, 1990[28]	Total hip arthroplasty	40	Tinzaparin, 50 anti-Xa / kg, 1x daily	2h before surgery, for 7 days	No intervention	Any VTE	NS
Roth, 1995[29]	Knee arthroscopy	122	Nadroparin, 2850 IU, 1x daily	2h before surgery, for 4 days	No intervention	Symptomatic VTE; major bleeding	6-8 weeks
Wirth, 2001[30]	Knee arthroscopy	239	Reviparin, 1750 IU, 1x daily	7 to 10 days	No intervention	Symptomatic VTE; major bleeding; any VTE	7-10 days
Yoo, 1997[31]	Total hip arthroscopy	100	Nadroparin, 2850-5700 IU, 1x daily	Start 12h before surgery, for 10 days	No intervention	Major bleeding; any VTE	10 days
**Ambulatory oncological patients**							
Agnelli, 2012[32]	Ambulatory cancer patients receiving chemotherapy	3,212	Semuloparin, 20mg, 1x daily	Median 3.5 months	Placebo	Mortality; symptomatic VTE; major bleeding; clinically relevant non-major bleeding; any VTE	1 year

Trial	Patient type	Sample size	Intervention	Duration of intervention	Control	Outcomes	Follow-up
Agnelli, 2009[33]	Ambulatory cancer patients receiving chemotherapy	1,166	Nadroparin, 3800 IU, 1x daily	Start at day 1 chemotherapy, for maximum 120 days	Placebo	Mortality; symptomatic VTE; major bleeding; any VTE	110-120 days
Haas, 2012 (TOPIC-I)[34]	Ambulatory metastatic breast cancer patients receiving chemotherapy	353	Certoparin, 3000 IU, 1x daily	6 months	Placebo	Mortality, symptomatic VTE; major bleeding; any VTE	6 months
Haas, 2012 (TOPIC-II)[34]	Ambulatory lung cancer patients receiving chemotherapy	547	Certoparin, 3000 IU, 1x daily	6 months	Placebo	Mortality; symptomatic VTE; major bleeding; any VTE	6 months
Lavau-Denes, 2012[35]	Advanced or metastatic cancer and central venous acces device	282	Dalteparin 2500 IU, nadroparin 2850 IU, or enoxaparin 4000 IU, 1x daily	Start within 6 days for 90 days	No intervention	Mortality; symptomatic VTE; any VTE	90 days
Monreal, 1996[36]	Cancer patients with port-a-cath	32	Dalteparin, 2500 IU, 1x daily	Start 2h before placing catheter, for 90 days	No intervention	Mortality; major bleeding; any VTE	90 days
Niers, 2007[37]	Hematologic malignancy patients receiving CVC	113	Nadroparin, 2850 IU, 1x daily	2h before insertion CVC, for 3 weeks	Placebo	Symptomatic VTE; major bleeding; clinically relevant non-major bleeding; any VTE	21 days
Weber, 2008[38]	Cancer patients admitted for palliative care	20	Nadroparin, 2850 IU or 3800 IU, weight based, 1x daily	NS	No intervention	Mortality; symptomatic VTE; major bleeding; any VTE	56 days
**Neurology**							
Elias, 1990[39]	Hemiplegic post-ischemic stroke	30	CY-222 15.000 anti-Xa, 1x daily	Start 48h after infarct, for 14 days	No intervention	Mortality; symptomatic VTE; major bleeding; any VTE	14 days
Sandset, 1990[40]	Acute ischemic stroke	103	Dalteparin, 3000-5500 IU, weight based, 1x daily	Until hospital discharge	Placebo	Major bleeding; any VTE	28 days
Wurm, 2004[41]	Subarachnoid haemorrhage	117	Enoxaparin 20mg, 1x daily	Within 72h, for 3 weeks	Placebo	Mortality; symptomatic VTE; major bleeding	1 year

CVC: central venous catheter; h: hours; NS: not stated; SAE: serious adverse events; VTE: venous thromboembolism

Table S3. Ongoing trials.

No ongoing trials on low dose low-molecular weight heparin versus placebo or no treatment were identified.

Table S4. Risk of bias assessment.

	**Risk of bias arising from the randomization process** ***(selection bias)***	**Risk of bias due to deviations from the intended interventions** ***(performance bias)***	**Bias due to missing outcome data** ***(attrition bias)***	**Risk of bias in measurement of the outcome** ***(ascertainment bias)***	**Risk of bias in selection of the reported result**
Adrichem 2017 (POT-KAST)	+	+/-	+	+	+
Adrichem 2017 (POT-CAST)	+	+/-	+	+	+
Agnelli 2009	+	+	+	+	+
Agnelli 2012	+	+	+	+	+
Baca 1997	+/-	-	+/-	-	+/-
Balas 1992	+/-	-	-	+	+/-
Bergqvist 1996	+/-	+/-	+	+	+/-
Bruntink 2017	+	+/-	-	+	+
Burrows 2001	+/-	+	+	+	+
Elias 1990	+/-	-	-	-	+/-
Dong 2018	+/-	-	-	+/-	+
Fraisse 2000	+/-	+/-	+/-	+	+
Fuji 2008a	+/-	-	+/-	+	+
Fuji 2008b	+/-	-	+/-	+	+
Haas 2012 (TOPIC-I)	+	+	+	+	+
Haas 2012 (TOPIC-II)	+	+	+	+	+
Jorgensen 2002	+	-	-	+	+/-
Kock 1995	+/-	-	+	-	+
Kujath 1993	+/-	-	+	+/-	+/-
Lassen 1991	+/-	+	+/-	+	+/-
Lassen 2002	+	+	+	+	+
Lavau-Denes 2012	+/-	+/-	+	-	+/-
Levine 1996	+	+/-	+	+	+
Xiao-Lin 2001	+/-	+/-	+	-	+/-
Mahe 2005	+	+	+	+	+
Marassi 1993	+/-	+/-	+	-	+/-
Melon 1991	+/-	+/-	+	+/-	+
Monreal 1996	+/-	+/-	+	-	+/-

Murugesan 2010	+	-	+	+	+
Niers 2007	+	+/-	+/-	+	+
Norberto Garcia 2013	+/-	+/-	+	+	+
Nurmohamed 1996	+	+	+/-	+	+
Ockelford 1989	+	-	+	+/-	+
Ozcan 2004	+/-	-	+/-	-	+/-
Pezzuoli 1990	+/-	+	+/-	-	+
Samama 1999	+	+	+/-	+	+
Sandset 1990	-	+	-	+	+
Sorensen 1990	-	+/-	+	+/-	+/-
Roth 1995	+/-	-	+/-	-	-
Valle 1988	+/-	+/-	+	+	+/-
Weber 2008	-	-	+	-	+/-
Wirth 2001	+/-	+/-	+	+	+/-
Wurm 2004	+	+/-	+	+	+/-
Yoo 1997	+/-	+/-	+/-	-	+/-

Review of authors' judgements about each risk of bias domain for each included study. '+' indicates low risk of bias; '+/-' indicates some concerns, '-' indicates high risk of bias.

Table S5. Sensitivity analysis: best-worse and worst-best case scenario's.

Outcome	Bias risk of trials	Trials (patients)	Best-worst case scenario Lost to follow-up with all events in control group	Worst-best case scenario Lost to follow-up with all events in intervention group
**Mortality**				
	Low risk	5 (4,976)	RR 1.00 (95% CI 0.90 to 1.12)	RR 1.05 (95% CI 0.94 to 1.18)
	All	23 (15,946)	RR 0.60 (95% CI 0.42 to 0.86)	RR 1.52 (95% CI 1.08 to 2.15)
**Symptomatic VTE**				
	Low risk	5 (4,980)	RR 0.33 (95% CI 0.14 to 0.80)	RR 1.35 (95% CI 0.60 to 3.03)
	All	25 (16,823)	RR 0.20 (95% CI 0.11 to 0.36)	RR 2.87 (95% CI 1.38 to 5.95)
**Major bleeding**				
	Low risk	5 (4,980)	RR 0.90 (95% CI 0.45 to 1.81)	RR 2.19 (95% CI 0.73 to 6.54)
	All	33 (13,641)	RR 0.30 (95% CI 0.14 to 0.63)	RR 4.07 (95% CI 1.95 to 8.48)

CI: confidence interval; RR: relative risk; VTE: venous thromboembolism.

Table S6. Sensitivity analysis: trials with publication year ≥ 2005.

Outcome	Trials	Patients	Effect estimate [a]
**Mortality**	10	8,149	RR 1.01 (95% CI 0.90 to 1.13)
**Symptomatic VTE**	12	8,286	RR 0.65 (95% CI 0.48 to 0.89)
**Major bleeding**	13	8,458	RR 1.44 (95% CI 0.68 to 3.03)
**SAE**	5	4,415	RR 0.91 (95% CI 0.70 to 1.19)
**Clinically relevant non-major bleeding**	3	2,999	RR 0.83 (95% CI 0.22 to 3.14)
**Any VTE**	10	2,170	RR 0.74 (95% CI 0.55 to 0.97)

CI: confidence interval; RR: relative risk; SAE: serious adverse events; VTE: venous thromboembolism.

Table S7. Sensitivity analysis: including LMWH types not a priori defined.

Outcome	Included trials	Trials (patients)	Conventional analysis	Trial sequential analysis[a] *α 2.5%; ß 90%; RRR 20%; D^2 model variance based*
**Mortality**				
	Low bias risk	6 (8,172)	RR 0.99 (0.93 to 1.06)	RR 0.99 (0.92 to 1.07)
	All	26 (18,973)	RR 0.99 (0.91 to 1.08)	RR 0.99 (0.92 to 1.07)
**VTE symptomatic**				
	Low bias risk	6 (8,090)	RR 0.50 (0.33 to 0.77)	RR 0.52 (0.11 to 2.57)
	All	27 (19,160)	RR 0.55 (0.43 to 0.70)	RR 0.55 (0.39 to 0.76)
**Major bleeding**				
	Low bias risk	6 (8,132)	RR 1.27 (0.77 to 2.09)	Insufficient data (<5% of DIS)
	All	36 (16,537)	RR 1.06 (0.76 to 1.48)	RR 1.02 (0.25 to 4.19)
**SAE**				
	Low bias risk	2 (4,362)	RR 1.01 (0.91 to 1.13)	RR 1.01 (0.80 to 1.28)
	All	9 (8,392)	RR 1.02 (0.92 to 1.14)	RR 1.02 (0.90 to 1.17) *FUTILITY CROSSED*
**Clinically relevant non-major bleeding**				
	Low bias risk	1 (3,172)	RR 1.85 (0.97 to 3.53)	Insufficient data (<5% of DIS)
	All	6 (6,544)	RR 1.69 (1.04 to 2.74)	Insufficient data (<5% of DIS)
**Any VTE**				
	Low bias risk	3 (1,254)	RR 0.57 (0.38 to 0.84)	RR 0.57 (0.11 to 2.82)
	All	32 (6,076)	RR 0.60 (0.49 to 0.72)	RR 0.62 (0.48 to 0.80)

α: two-sided significance level, ß: power; D^2: diversity; DIS: diversity adjusted information size; OR: odds ratio; RR: relative risk; RRR: relative risk reduction; SAE: serious adverse events; TSA: trial sequential analysis; VTE: venous thromboembolism; [a] Small discrepancies of the intervention effect estimates between the traditional RevMan meta-analyses and the TSA adjusted results may occur due to different pooling methods (for example the inclusion of zero-event trials in TSA analyses)

Table S8. Subgroup analysis: co-primary outcomes.

Subgroup	Trials	Patients	Effect estimate [a] Risk ratio (95% confidence interval)	Test of interaction P-value
**All-cause mortality**				
**Risk of bias**	23	15,487	0.99 (0.85 to 1.14)	0.39
Low risk of bias	5	4,960	1.03 (0.92 to 1.16)	
High risk of bias	18	10,527	0.87 (0.60 to 1.26)	
**Population**	23	15,487	0.99 (0.85 to 1.14)	0.95
Acutely ill medical	3	3,408	1.00 (0.83 to 1.22)	
Surgery	6	5,584	0.66 (0.18 to 2.41)	
Orthopedics or immobilisation	6	3,905	0.64 (0.08 to 5.17)	
Oncology	6	2,370	1.01 (0.89 to 1.16)	
Neurology	2	220	1.46 (0.16 to 13.13)	
**LMWH type**	23	15,487	0.99 (0.85 to 1.14)	0.97
Enoxaparin	2	830	1.02 (0.72 to 1.45)	
Dalteparin	5	664	0.90 (0.31 to 2.62)	
Nadroparin	7	9,034	0.96 (0.74 to 1.26)	
Tinzaparin	3	475	0.50 (0.07 to 3.82)	
Certoparin	2	898	0.98 (0.73 to 1.32)	
Reviparin	1	438	Not estimable	
Bemiparin	1	262	Not estimable	
Parnaparin	0	0	Not estimable	
Various	2	2,886	0.33 (0.01 to 8.13)	
**Length of intervention**	23	15,487	0.99 (0.85 to 1.14)	0.92
Intervention period > 30 days	6	1,824	0.96 (0.72 to 1.27)	
Intervention period < 30 days	17	13,663	0.98 (0.80 to 1.20)	
**Length of follow-up**	23	15,487	1.01 (0.91 to 1.12) [b]	0.55
Follow-up > 30 days	10	3,467	1.07 (0.86 to 1.32) [b]	
Follow-up < 30 days	12	11,815	0.99 (0.88 to 1.12) [b]	
Follow-up unclear	1	205	Not estimable	
**Symptomatic venous thromboembolism**				
**Risk of bias**	25	15,920	0.62 (0.48 to 0.81)	0.75
Low risk of bias	5	4,878	0.65 (0.45 to 0.94)	
High risk of bias	20	11,042	0.59 (0.40 to 0.88)	
**Population**	25	15,920	0.62 (0.48 to 0.81)	0.37
Acutely ill medical	3	3,038	0.86 (0.56 to 1.34)	
Surgery	7	6,153	0.45 (0.22 to 0.91)	
Orthopedics or immobilisation	8	4,199	0.61 (0.32 to 1.15)	
Oncology	6	2,413	0.47 (0.28 to 0.78)	
Neurology	1	117	1.05 (0.07 to 16.43)	

Subgroup	Trials	Patients	Effect estimate [a] Risk ratio (95% confidence interval)	Test of interaction P-value
**LMWH type**	25	15,920	0.62 (0.48 to 0.81)	0.69
Enoxaparin	2	643	1.41 (0.45 to 4.41)	
Dalteparin	3	387	0.85 (0.12 to 5.78)	
Nadroparin	10	9,136	0.58 (0.41 to 0.82)	
Tinzaparin	2	395	0.35 (0.01 to 8.42)	
Certoparin	2	883	0.57 (0.24 to 1.35)	
Reviparin	3	1,328	0.34 (0.05 to 2.50)	
Bemiparin	1	262	Not estimable	
Parnaparin	0	0	Not estimable	
Various	2	2,886	0.92 (0.45 to 1.88)	
**Length of intervention**	25	15,920	0.62 (0.48 to 0.81)	0.07
Intervention period > 30 days	6	2,863	0.42 (0.25 to 0.70)	
Intervention period < 30 days	19	13,057	0.73 (0.53 to 1.00)	
**Length of follow-up**	25	15,920	0.62 (0.48 to 0.81)	0.93
Follow-up > 30 days	16	7,255	0.63 (0.44 to 0.91)	
Follow-up < 30 days	7	8,271	0.62 (0.41 to 0.93)	
Follow-up unclear	2	394	0.34 (0.01 to 8.17)	
**Major bleeding**				
**Risk of bias**	33	13,091	1.07 (0.72 to 1.59)	0.18
Low risk of bias	5	4,960	1.70 (0.77 to 3.74)	
High risk of bias	28	8,131	0.91 (0.58 to 1.45)	
**Population**	33	13,091	1.07 (0.72 to 1.59)	0.30
Acutely ill medical	3	3,408	0.87 (0.32 to 2.38)	
Surgery	10	2,313	0.98 (0.51 to 1.87)	
Orthopedics or immobilisation	12	4,940	0.95 (0.23 to 3.92)	
Oncology	6	2,210	2.21 (0.96 to 5.09)	
Neurology	2	220	0.45 (0.12 to 1.66)	
**LMWH type**	33	13,091	1.07 (0.72 to 1.59)	0.38
Enoxaparin	5	1,339	0.52 (0.23 to 1.18)	
Dalteparin	4	391	1.08 (0.31 to 3.75)	
Nadroparin	12	5,196	1.67 (0.71 to 3.93)	
Tinzaparin	2	285	3.15 (0.13 to 75.08)	
Certoparin	3	1,237	1.93 (0.75 to 4.98)	
Reviparin	3	1,395	0.83 (0.36 to 1.92)	
Bemiparin	1	262	Not estimable	
Parnaparin	1	100	Not estimable	
Various	2	2,886	0.98 (0.06 to 15.72)	
**Length of intervention**	33	13,091	1.07 (0.72 to 1.59)	0.04
Intervention period > 30 days	7	2,721	2.20 (1.00 to 4.82)	
Intervention period < 30 days	26	10,370	0.84 (0.53 to 1.32)	
**Length of follow-up**	33	13,091	1.07 (0.72 to 1.59)	0.49
Follow-up > 30 days	18	7,944	1.18 (0.72 to 1.93)	
Follow-up < 30 days	11	4,607	0.96 (0.48 to 1.91)	

Subgroup	Trials	Patients	**Effect estimate** [a] Risk ratio (95% confidence interval)	**Test of interaction** P-value
Follow-up unclear	4	540	0.20 (0.01 to 4.15)	

[a] Meta-analyses were performed using a random-effects model unless stated otherwise; [b] Meta-analysis performed using a fixed effect model.

Table S9. Sensitivity analysis: proportion of SAE and cumulative SAE.

Outcome	Trials (patients)	SAE – proportion	SAE – cumulative
**SAE**	37 (18,688)	RR 0.96 (95% CI 0.87 to 1.07)	RR 0.94 (95% CI 0.87 to 1.02)

CI: confidence interval; RR: relative risk; SAE: serious adverse events.

Study or Subgroup	LMWH Events	LMWH Total	Control Events	Control Total	Weight	Risk Ratio M-H, Random, 95% CI
**4.2.1 Acutely ill medical patients**						
Fraisse 2000	27	108	22	113	22.5%	1.28 [0.78, 2.11]
**Subtotal (95% CI)**		**108**		**113**	**22.5%**	**1.28 [0.78, 2.11]**
Total events	27		22			
Heterogeneity: Not applicable						
Test for overall effect: Z = 0.99 (P = 0.32)						
**4.2.2 Surgery**						
**Subtotal (95% CI)**		**0**		**0**		**Not estimable**
Total events	0		0			
Heterogeneity: Not applicable						
Test for overall effect: Not applicable						
**4.2.3 Orthopedics or immobilization**						
Adrichem 2017 (POT-CAST)	2	719	0	716	0.6%	4.98 [0.24, 103.53]
Adrichem 2017 (POT-KAST)	0	731	0	720		Not estimable
Fuji 2008a	0	100	1	101	0.5%	0.34 [0.01, 8.17]
Fuji 2008b	2	89	1	89	1.0%	2.00 [0.18, 21.66]
Jorgensen 2002	0	99	0	106		Not estimable
Kock 1995	0	176	0	163		Not estimable
**Subtotal (95% CI)**		**1914**		**1895**	**2.1%**	**1.64 [0.33, 8.25]**
Total events	4		2			
Heterogeneity: Tau² = 0.00; Chi² = 1.49, df = 2 (P = 0.47); I² = 0%						
Test for overall effect: Z = 0.60 (P = 0.55)						
**4.2.4 Oncology**						
Agnelli 2009	121	769	67	381	75.3%	0.89 [0.68, 1.17]
**Subtotal (95% CI)**		**769**		**381**	**75.3%**	**0.89 [0.68, 1.17]**
Total events	121		67			
Heterogeneity: Not applicable						
Test for overall effect: Z = 0.80 (P = 0.42)						
**4.2.5 Neurology**						
**Subtotal (95% CI)**		**0**		**0**		**Not estimable**
Total events	0		0			
Heterogeneity: Not applicable						
Test for overall effect: Not applicable						
**Total (95% CI)**		**2791**		**2389**	**100.0%**	**0.98 [0.78, 1.25]**
Total events	152		91			
Heterogeneity: Tau² = 0.00; Chi² = 3.44, df = 4 (P = 0.49); I² = 0%						
Test for overall effect: Z = 0.14 (P = 0.89)						
Test for subgroup differences: Chi² = 1.95, df = 2 (P = 0.38), I² = 0%						

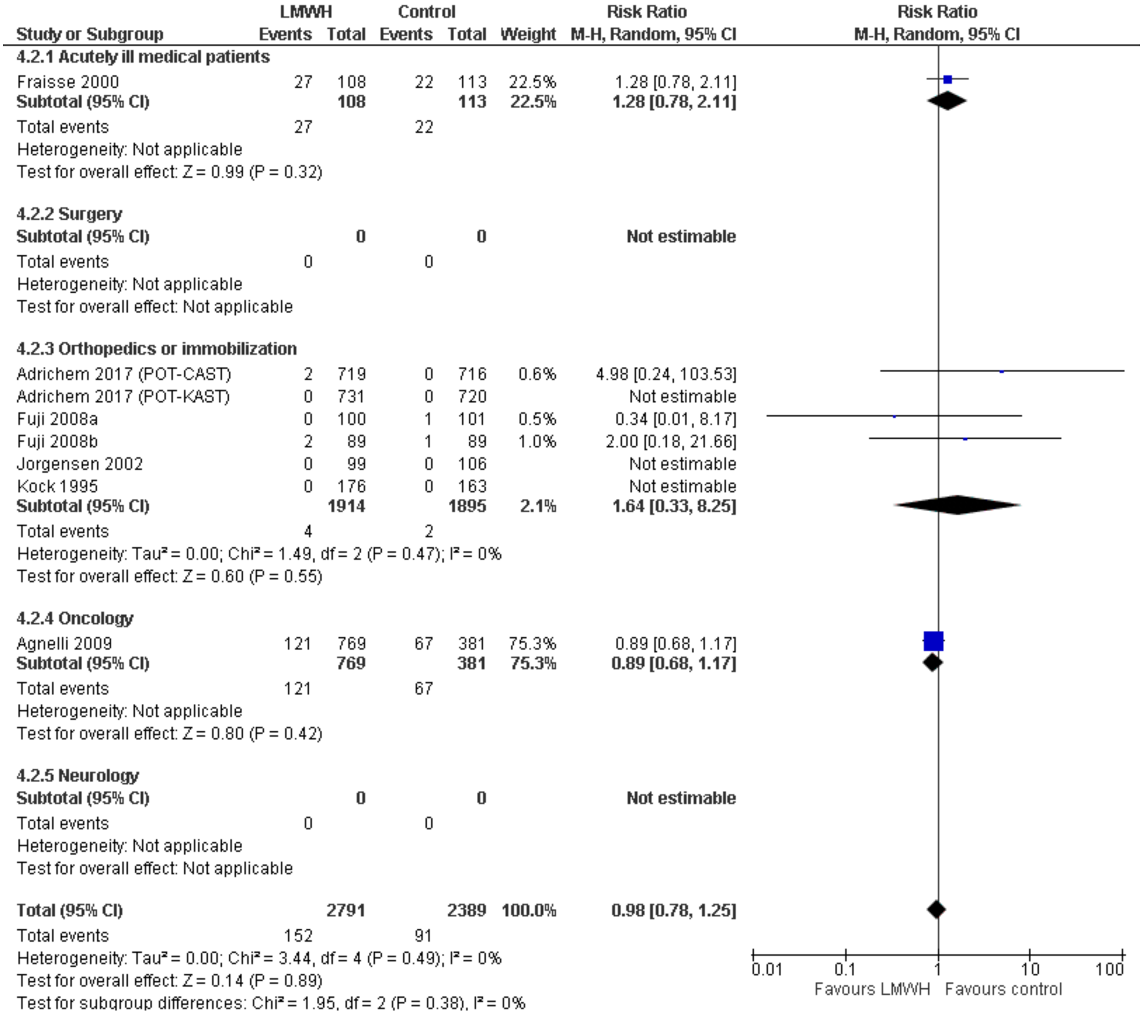

Figure S1. Forest plot of SAE, stratified for patient type. Forest plot of Serious Adverse Events (SAE) at maximal follow-up of LMWH prophylaxis compared to placebo or no treatment, stratified according to patient type. Size of the squares reflects the weight of the trial in the pooled analysis. Horizontal bars represent 95% confidence intervals. LMWH: Low-molecular-weight heparin; SAE: Serious Adverse Events; VTE: venous thromboembolism.

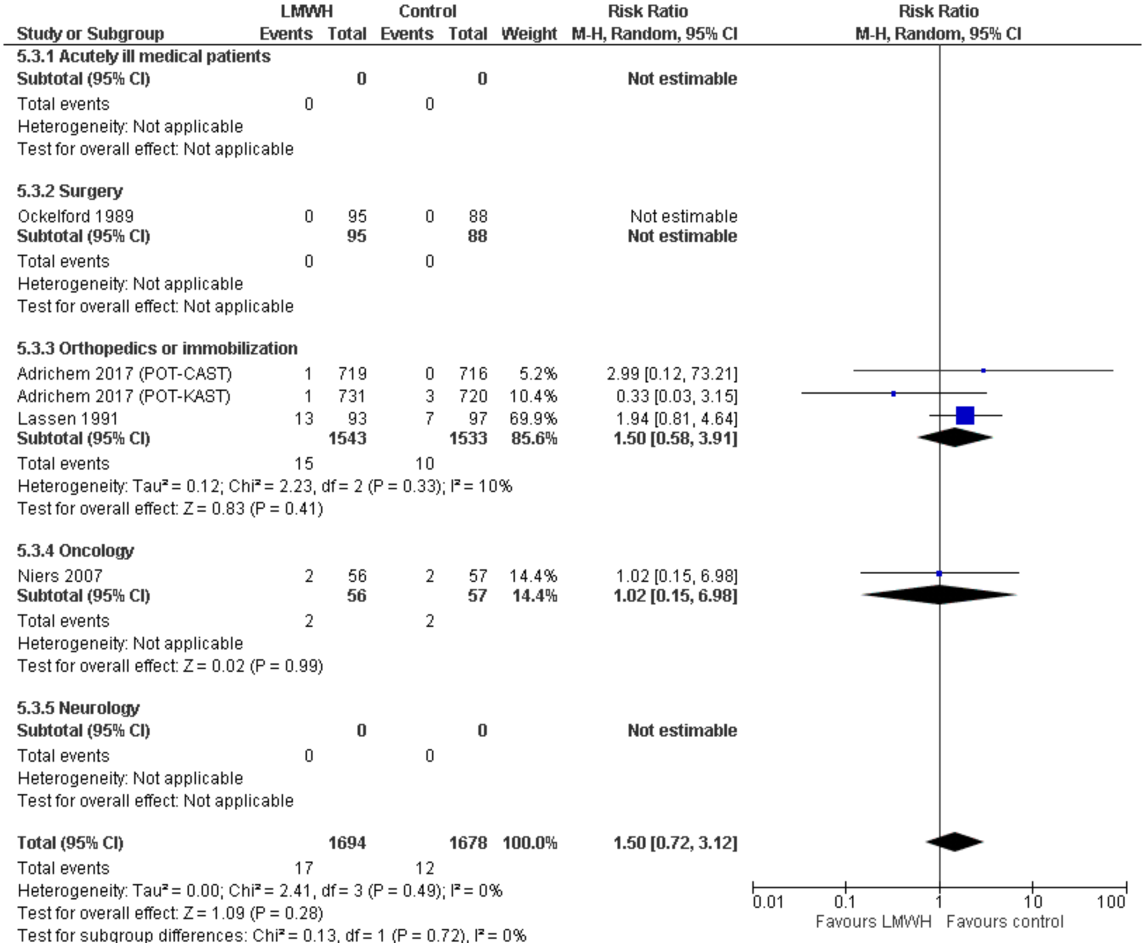

Figure S2. Forest plot of clinically relevant non-major bleeding, stratified for patient type; Forest plot of clinically relevant non-major bleeding at maximal follow-up of LMWH prophylaxis compared to placebo or no treatment, stratified according to patient type. Size of the squares reflects the weight of the trial in the pooled analysis. Horizontal bars represent 95% confidence intervals. LMWH: Low-molecular-weight heparin; VTE: venous thromboembolism.

Study or Subgroup	LMWH Events	LMWH Total	Control Events	Control Total	Weight	Risk Ratio M-H, Random, 95% CI
**6.2.1 Acutely ill medical patients**						
Fraisse 2000	13	84	24	85	5.1%	0.55 [0.30, 1.00]
Samama 1999	46	263	45	263	7.2%	1.02 [0.70, 1.49]
**Subtotal (95% CI)**		**347**		**348**	**12.3%**	**0.78 [0.43, 1.43]**
Total events	59		69			
Heterogeneity: Tau² = 0.13; Chi² = 2.96, df = 1 (P = 0.09); I² = 66%						
Test for overall effect: Z = 0.79 (P = 0.43)						
**6.2.2 Surgery**						
Balas 1992	1	94	8	95	0.8%	0.13 [0.02, 0.99]
Bergqvist 1996	3	39	10	41	2.1%	0.32 [0.09, 1.06]
Dong 2018	9	40	8	50	3.4%	1.41 [0.60, 3.31]
Marassi 1993	2	30	11	31	1.6%	0.19 [0.05, 0.78]
Melon 1991	10	64	14	58	4.2%	0.65 [0.31, 1.34]
Murugesan 2010	0	34	0	31		Not estimable
Norberto Garcia 2013	0	130	0	132		Not estimable
Nurmohamed 1996	33	166	51	179	7.1%	0.70 [0.48, 1.02]
Ockelford 1989	4	95	14	88	2.5%	0.26 [0.09, 0.77]
Valle 1988	0	50	3	50	0.4%	0.14 [0.01, 2.70]
**Subtotal (95% CI)**		**742**		**755**	**22.2%**	**0.49 [0.30, 0.81]**
Total events	62		119			
Heterogeneity: Tau² = 0.22; Chi² = 13.89, df = 7 (P = 0.05); I² = 50%						
Test for overall effect: Z = 2.78 (P = 0.005)						
**6.2.3 Orthopedics or immobilization**						
Bruntink 2017	2	92	11	94	1.5%	0.19 [0.04, 0.82]
Fuji 2008a	21	81	36	86	6.5%	0.62 [0.40, 0.97]
Fuji 2008b	35	78	48	79	7.9%	0.74 [0.55, 1.00]
Jorgensen 2002	10	99	18	106	4.2%	0.59 [0.29, 1.23]
Kock 1995	0	176	7	163	0.5%	0.06 [0.00, 1.07]
Kujath 1993	6	126	21	127	3.3%	0.29 [0.12, 0.69]
Lassen 1991	30	93	45	97	7.3%	0.70 [0.48, 1.00]
Lassen 2002	17	183	35	188	5.6%	0.50 [0.29, 0.86]
Sorensen 1990	11	20	11	20	5.5%	1.00 [0.57, 1.75]
Wirth 2001	1	117	5	122	0.8%	0.21 [0.02, 1.76]
Xiao-Lin 2001	1	23	8	23	0.9%	0.13 [0.02, 0.92]
Yoo 1997	1	50	8	50	0.8%	0.13 [0.02, 0.96]
**Subtotal (95% CI)**		**1138**		**1155**	**44.9%**	**0.55 [0.41, 0.73]**
Total events	135		253			
Heterogeneity: Tau² = 0.10; Chi² = 21.38, df = 11 (P = 0.03); I² = 49%						
Test for overall effect: Z = 4.06 (P < 0.0001)						
**6.2.4 Oncology**						
Haas 2012 (TOPIC I)	7	174	7	177	2.7%	1.02 [0.36, 2.84]
Haas 2012 (TOPIC II)	12	268	22	264	4.5%	0.54 [0.27, 1.06]
Lavau-Denes 2012	14	138	20	135	4.8%	0.68 [0.36, 1.30]
Monreal 1996	1	16	8	13	0.9%	0.10 [0.01, 0.71]
Niers 2007	7	41	4	46	2.2%	1.96 [0.62, 6.23]
**Subtotal (95% CI)**		**637**		**635**	**15.1%**	**0.71 [0.39, 1.30]**
Total events	41		61			
Heterogeneity: Tau² = 0.22; Chi² = 8.01, df = 4 (P = 0.09); I² = 50%						
Test for overall effect: Z = 1.12 (P = 0.26)						
**6.2.5 Neurology**						
Sandset 1990	15	42	17	50	5.5%	1.05 [0.60, 1.84]
**Subtotal (95% CI)**		**42**		**50**	**5.5%**	**1.05 [0.60, 1.84]**
Total events	15		17			
Heterogeneity: Not applicable						
Test for overall effect: Z = 0.17 (P = 0.86)						
**Total (95% CI)**		**2906**		**2943**	**100.0%**	**0.61 [0.50, 0.75]**
Total events	312		519			
Heterogeneity: Tau² = 0.10; Chi² = 51.19, df = 27 (P = 0.003); I² = 47%						
Test for overall effect: Z = 4.89 (P < 0.00001)						
Test for subgroup differences: Chi² = 5.66, df = 4 (P = 0.23), I² = 29.3%						

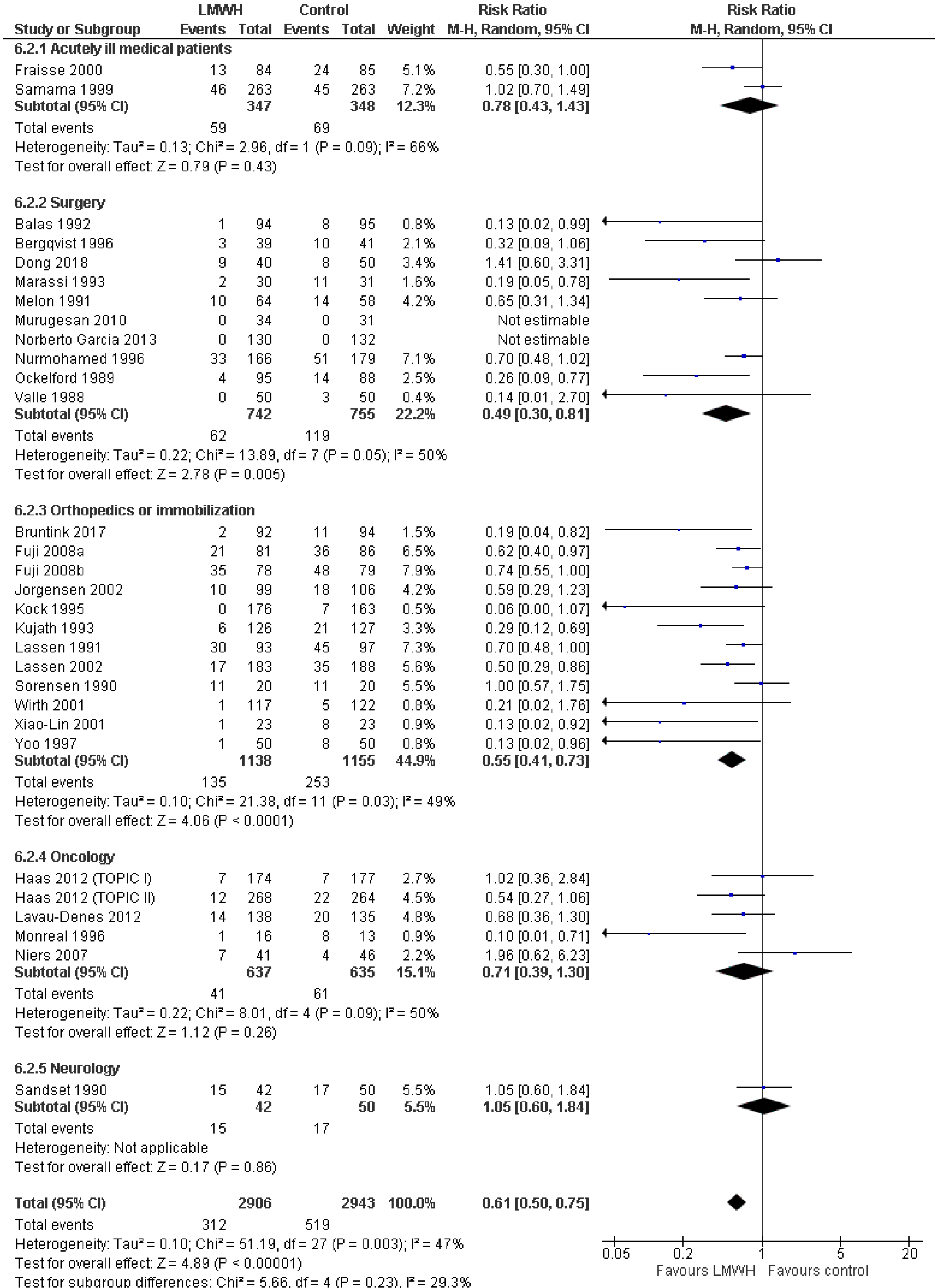

Figure S3. Forest plot of any VTE, stratified for patient type; Forest plot of any VTE at maximal follow-up of LMWH prophylaxis compared to placebo or no treatment, stratified according to patient type. Size of the squares reflects the weight of the trial in the pooled analysis. Horizontal bars represent 95% confidence intervals. LMWH: Low-molecular-weight heparin; VTE: venous thromboembolism.

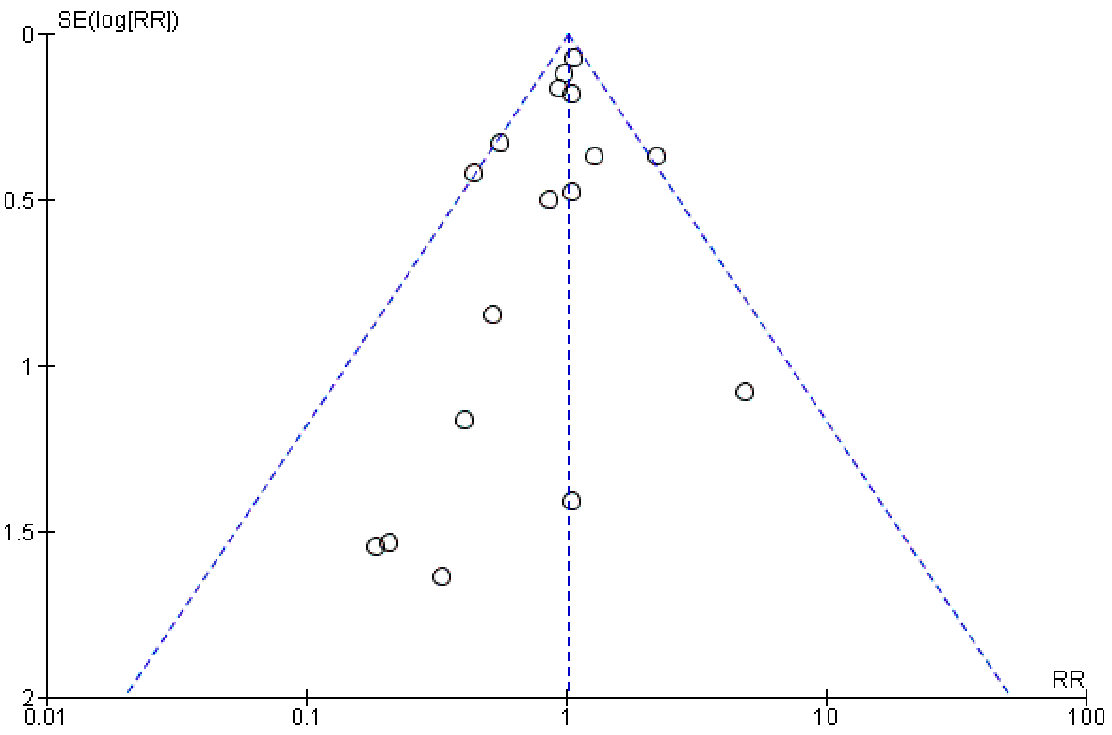

Figure S4. Funnel plot of all-cause mortality.

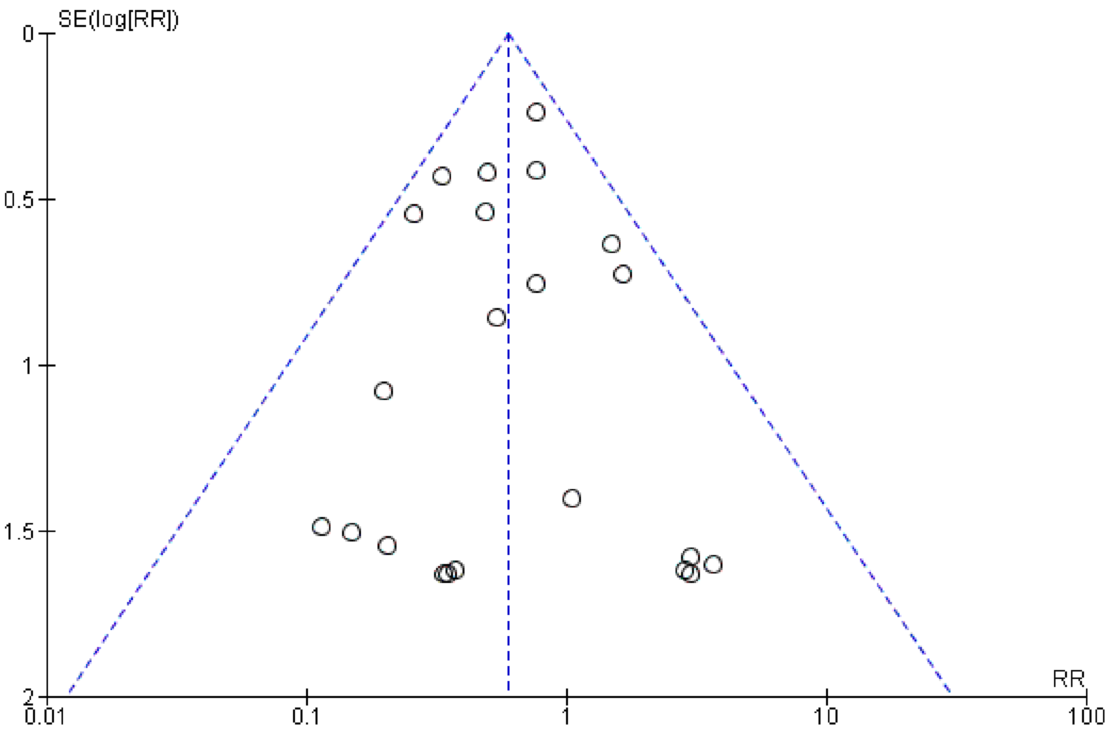

Figure S5. Funnel plot of symptomatic VTE.

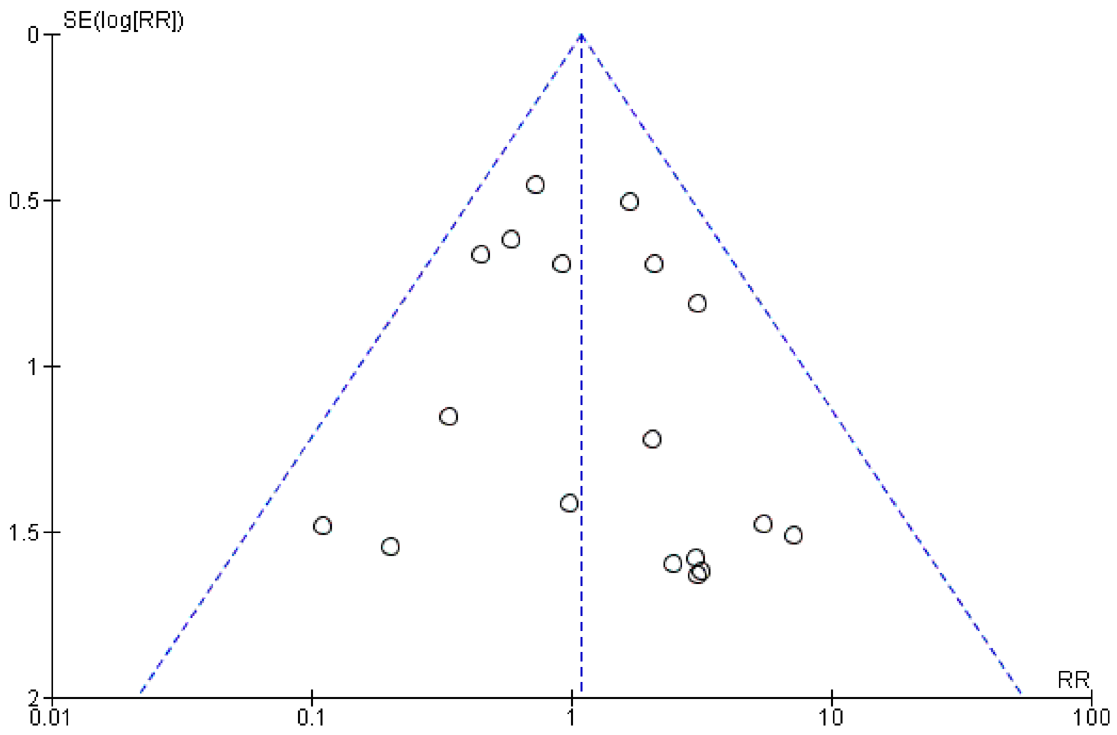

Figure S6. Funnel plot of major bleeding.

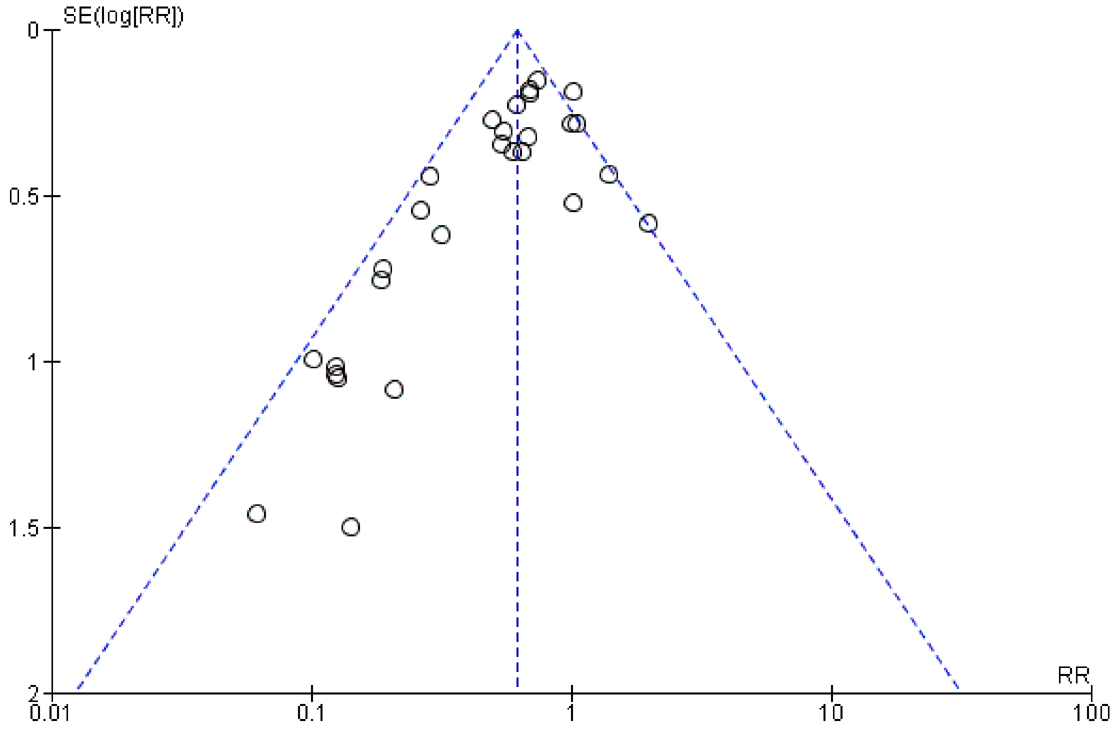

Figure S7. Funnel plot of any VTE

References

1. Fraisse, F.; Holzapfel, L.; Couland, J.M.; Simonneau, G.; Bedock, B.; Feissel, M.; Herbecq, P.; Pordes, R.; Poussel, J.F.; Roux, L. Nadroparin in the prevention of deep vein thrombosis in acute decompensated COPD. *Am. J. Respir. Crit. Care Med.* **2000**, *161*, 1109–1114.
2. Mahé, I.; Bergmann, J.F.; D'Azémar, P.; Vaissie, J.J.; Caulin, C. Lack of effect of a low-molecular-weight heparin (nadroparin) on mortality in bedridden medical in-patients: A prospective randomised double-blind study. *Eur. J. Clin. Pharmacol.* **2005**, *61*, 347–351.
3. Ozcan, M.; Soydan, E.; Ustun, C.; Celebi, H.; Arslan, O.; Arat, M.; Gurman, G.; Ilhan, O.; Koc, N.; Konuk, A.; et al. The use of low molecular weight heparins (dalteparin) for prophylaxis against thromboembolic complications during allogeneic haematopoetic stem cell transplantation [Abstract]. In Proceedings of the EBMT; 2004; pp. 303–360.
4. Samama, M.M.; Cohen, A.T.; Darmon, J.Y.; Desjardins, L.; Eldor, A.; Janbon, C.; Leizorovicz, A.; Nguyen, H.; Olsson, C.G.; Turpie, A.G.; et al. A comparison of enoxaparin with placebo for the prevention of venous thromboembolism in acutely ill medical patients. *N. Engl. J. Med.* **1999**, *341*, 793–800.
5. Baca, I.; Schneider, B.; Kohler, T.; Misselwitz, F.; Zehle, A.; Muhe, F. Thromboembolieprophylaxe bei minimal-invasiven Eingriffen und kurzstationarer Behandlung. *Der Chir.* **1997**, *68*, 1275–1280.
6. Balas, P.E.; Athens, G.; Sanofi Recherche, F. Efficacy and safety of nadroparin (fraxiparine) versus placebo in the prophylactic treatment of deep vein thrombosis in patients with high thrombo-embolic risk undergoing general surgery. *Thromb. Res.* **2004**, *65*, S113.
7. Bergqvist, D.; Flordal, P.A.; Friberg, B.; Frisell, J.; Hedberg, M.; Ljungstrom, K.G.; Matzsch, T.; Torngren, S. Thromboprophylaxis with a low molecular weight heparin (Tinzaparin) in emergency abdominal surgery. *Zeitschrift fur Geffasskrankheiten* **1996**, *25*.
8. Burrows, R.F.; Gan, E.T.; Gallus, A.S.; Wallace, E.M.; Burrows, E.A. A randomised double-blind placebo controlled trial of low molecular weight heparin as prophylaxis in preventing venous thrombolic events after caesarean section: A pilot study. *Br. J. Obstet. Gynaecol.* **2001**, *108*, 835–839.
9. Dong, J.; Wang, J.; Feng, Y.; Li-Ping, Q.; Fang, H.; Wang, G.-D.; Wu, Z.-Q.; Wang, H.-Z.W.; Yang, Y.; Li, Q. Effect of low molecular weight heparin on venous thromboembolism disease in thoracotomy patients with cancer. *J. Thorac. Dis.* **2018**, *10*, 1850–1856.
10. Marassi, A.; Balzano, G.; Mari, G.; Vigano D'Angelo, S.; Della Valle, P.; Di Carlo, V.; D'Angelo, A. Prevention of Postoperative Deep Vein Thrombosis in Cancer Patients. A Randomized Trial with Low Molecular Weight Heparin (CY 216). *Int. Surg.* **1993**, *78*, 166–170.
11. Melon, E.; Keravel, Y.; Gaston, A.; Huet, Y.; Combes, S. Deep venous thrombosis prophylaxis by low molecular weight heparin in neurosurgical patients [abstract]. *Anesthesiology* **1991**, *75*.
12. Murugesan, A.; Srivastava, D.N.; Ballehaninna, U.K.; Chumber, S.; Dhar, A.; Misra, M.C.; Parshad, R.; Seenu, V.; Srivastava, A.; Gupta, N.P. Detection and Prevention of Post-Operative Deep Vein Thrombosis [DVT] Using Nadroparin Among Patients Undergoing Major Abdominal Operations in India; a Randomised Controlled Trial. *Indian J. Surg.* **2010**, *72*, 312–317.
13. Norberto Garcia, E.M.S.; Merino, B.; Taylor, J.H.; Vizcaino, I.; Vaquero, C. Low-Molecular-Weight Heparin for prevention fo venous thromboembolism after varicose vein surgery in moderate-risk patients: a randomized, controlled trial. *Ann. Vasc. Surg.* **2013**, *27*, 940–946.
14. Nurmohamed, M.T.; Van Riel, A.M.; Henkens, C.M.A.; Koopman, M.M.W.; Que, G.T.H.; D'Azemar, P.; Büller, H.R.; Ten Cate, J.W.; Hoek, J.A.; Van Der Meer, J.; et al. Low molecular weight heparin and compression stockings in the prevention of venous thromboembolism in neurosurgery. *Thromb. Haemost.* **1996**, *75*, 233–238.
15. Ockelford, P.A.; Patterson, J.; Johns, A.S. A double-blind randomized placebo controlled trial of thromboprophylaxis in major elective general surgery using once daily injections of a low molecular weight heparin fragment (Fragmin). *Thromb. Haemost.* **1989**, *62*, 1046–1049.
16. Pezzuoli, G.; Serneri, G.G.N.; Settembrini, P.G.; Coggi, G.; Olivari, N.; Negri, G.; Codemo, R.; Galli, G.; Roveri, S.; Negri, G.; et al. Effectiveness and safety of the low-molecular-weight heparin cy 216 in the prevention of fatal pulmonary embolism and thromboembolic death in general surgery: A multicentre, double-blind, randomized controlled clinical trial versus placebo (step). *Haemostasis* **1990**, *20*, 193–204.
17. Valle, I.; Sola, G.; Origone, A. Controlled clinical study of the efficacy of a new low molecular weight heparin administered subcutaneously to prevent post-operative deep venous thrombosis. *Curr. Med. Res. Opin.* **1988**, *11*, 80–86.
18. van Adrichem, R.A.; Nemeth, B.; Algra, A.; le Cessie, S.; Rosendaal, F.R.; Schipper, I.B.; Nelissen, R.G.H.H.; Cannegieter, S.C. Thromboprophylaxis after Knee Arthroscopy and Lower-Leg Casting. *N.*

Engl. J. Med. **2017**, *376*, 515–525.
19. Bruntink, M.M.; Groutars, Y.M.E.; Schipper, I.B.; Breederveld, R.S.; Tuinebreijer, W.E.; Derksen, R.J.; America, O.W.M.; van den Berg, W.; Bevort, A.H.; Bilars, P.M.; et al. Nadroparin or fondaparinux versus no thromboprophylaxis in patients immobilised in a below-knee plaster cast (PROTECT): A randomised controlled trial. *Injury* **2017**, *48*, 936–940.
20. Fuji, T.; Ochi, T.; Niwa, S.; Fujita, S. Prevention of postoperative venous thromboembolism in Japanese patients undergoing total hip or knee arthroplasty: Two randomized, double-blind, placebo-controlled studies with three dosage regimens of enoxaparin. *J. Orthop. Sci.* **2008**, *13*, 442–451.
21. Jorgensen, P.; Knudsen, J.B.; Broeng, L.; Josephsen, L.; Bjerregaard, P.; Hagen, K.; Jorgensen, P.; Torholm, C. The thromboprophylactic effect of a Low-Molecular-Weight Heparin (Fragmin) in Hip Fracture Surgery. A Placebo-Controlled Study. *Clin. Orthop. Relat. Res.* **1992**, 95–100.
22. Kock, H.J.; Schmit-Neuerburg, K.P.; Hanke, J.; Rudofsky, G.; Hirsche, H. Thromboprophylaxis with low-molecular-weight heparin in outpatients with plaster-cast immobilisation of the leg. *Lancet* **1995**, *346*, 459–461.
23. Kujath, P.; Spannagel, U.; Habscheid, W. Incidence and prophylaxis of deep venous thrombosis in outpatients with injury of the lower limb. *Haemostasis* **1993**, *23 Suppl 1*, 20–26.
24. Lassen, M.R.; Borris, L.C.; Christiansen, H.M.; Boll, K.L.; Eiskjær, S.P.; Nielsen, B.W.; Schøstt, P.; Olsen, A.D.; Rodenberg, J.C.; Lucht, U. Prevention of thromboembolism in 190 hip arthroplasties: Comparison of LMW heparin and placebo. *Acta Orthop.* **1991**, *62*, 33–38.
25. Lassen, M.R.; Borris, L.C.; Nakov, R.L. Use of the low-molecular-weight heparin reviparin to prevent deep-vein thrombosis after leg injury requiring immolbilization. *N. Engl. J. Med.* **2002**, *347*, 726–730.
26. Levine, M.N.; Gent, M.; Hirsh, J.; Weitz, J.; Turpie, A.G.; Powers, P.; Neemeh, J.; Willan, A.; Skingley, P. Ardeparin (low-molecular-weight heparin) vs graduated compression stockings for the prevention of venous thromboembolism. A randomized trial in patients undergoing knee surgery. *Arch. Intern. Med.* **1996**, *156*, 851–6.
27. Xiao-Lin, L.; Wei-Jie, L.; Nan-Sheng, Y. Prophylaxis for deep vein thrombosis with low molecular weight heparin following hip and knee surgery. *Chinese J reparative Reconstr. Surg.* **2001**, *15*, 39–41.
28. Sørensen, J. V.; Lassen, M.R.; Borris, L.C.; Jørgensen, P.S.; Schøtt, P.; Weber, S.; Murphy, R.; Walenga, J. Association between plasma levels of tissue plasminogen activator and postoperative deep vein thrombosis -influence of prophylaxis with a low molecular weight heparin. *Thromb. Res.* **1990**, *60*, 247–251.
29. Roth, V.P. Prophylaxis of deep vein thrombosis in outpatients undergoing arthroscopic meniscus operation. *Orthop. Prax.* **1995**, *5*, 345–347.
30. Wirth, T.; Schneider, B.; Misselwitz, F.; Lomb, M.; Tüylü, H.; Egbring, R.; Griss, P. Prevention of venous thromboembolism after knee arthroscopy with low-molecular weight heparin (Reviparin): Results of a randomized controlled trial. *Arthroscopy* **2001**, *17*, 393–399.
31. Yoo; C., M.; Kang; S., C.; Kim; H., Y.; Kim; K., S. A prospective randomized study on the use of nadroparin calcium in the prophylaxis of thromboembolism in Korean patients undergoing elective total hip replacement. *Int. Orthop.* **1997**, *21*, 399–402.
32. Agnelli, G.; George, D.J.; Kakkar, A.K.; Fisher, W.; Lassen, M.R.; Mismetti, P.; Mouret, P.; Chaudhari, U.; Lawson, F.; Turpie, A.G.G.; et al. Semuloparin for thromboprophylaxis in patients receiving chemotherapy for cancer. *N. Engl. J. Med.* **2012**, *366*, 601–609.
33. Agnelli, G.; Gussoni, G.; Bianchini, C.; Verso, M.; Mandala, M.; Cavanna, L.; Barni, S.; Labianca, R.; Buzzi, F.; Scambia, G.; et al. Nadroparin for the prevention of thromboembolic events in ambulatory patients with metastatic or locally advanced solid cancer receiving chemotherapy: a randomised, placebo-controlled, double-blind study. *Lancet. Oncol.* **2009**, *10*, 943–949.
34. Haas, S.K.; Freund, M.; Heigener, D.; Heilmann, L.; Kemkes-Matthes, B.; Tempelhoff, G.-F. von; Melzer, N.; Kakkar, A.K.; TOPIC Investigators; von Tempelhoff, G.-F.; et al. Low-Molecular-Weight Heparin Versus Placebo for the Prevention of Venous Thromboembolism in Metastatic Breast Cancer or Stage III/IV Lung Cancer. *Clin. Appl. Thromb. Hemost.* **2012**, *18*, 159–165.
35. Lavau-Denes, S.; Lacroix, P.; Maubon, A.; Preux, P.M.; Genet, D.; Vénat-Bouvet, L.; Labourey, J.L.; Martin, J.; Slaouti, P.; Tubiana-Mathieu, N. Prophylaxis of catheter-related deep vein thrombosis in cancer patients with low-dose warfarin, low molecular weight heparin, or control: A randomized, controlled, phase III study. *Cancer Chemother. Pharmacol.* **2013**, *72*, 65–73.
36. Monreal, M.; Alastrue, A.; Miquel, R.; Mira, X.; Muxart, J.; Rosell, R.; Abad, A. Upper Extremity Deep Venous Thrombosis in Cancer Patients with Venous Access Devices - Prophylaxis with a Low Molecular Weight Heparin (Fragmin). *Thromb. Haemost.* **1996**, *75*, 251–253.

37. Niers, T.M.H.; Di Nisio, M.; Klerk, C.P.W.; Baarslag, H.J.; Büller, H.R.; Biemond, B.J. Prevention of catheter-related venous thrombosis with nadroparin in patients receiving chemotherapy for hematologic malignancies: A randomized, placebo-controlled study. *J. Thromb. Haemost.* **2007**, *5*, 1878–1882.
38. Weber, C.; Merminod, T.; Herrmann, F.R.; Zulian, G.B. Prophylactic anti-coagulation in cancer palliative care: A prospective randomised study. *Support. Care Cancer* **2008**, *16*, 847–852.
39. Elias, A.; Milandre, L.; Lagrange, G.; Aillaud, M.F.; Alonzo, B.; Toulemonde, F.; Juhan-Vague, I.; Khalil, R.; Bayrou, B.; Serradimigni, A. Prevention of deep venous thrombosis of the leg by a very low molecular weight heparin fraction (CY 222) in patients with hemiplegia following cerebral infarction: a randomized pilot study (30 patients)]. *La Rev. Med. interne* **1989**, *11*, 95–8.
40. Sandset, P.M.; Dahl, T.; Stiris, M.; Rostad, B.; Scheel, B.; Abildgaard, U. A double-blind and randomized placebo-controlled trial of low molecular weight heparin once daily to prevent deep-vein thrombosis in acute ischemic stroke. *Semin. Thromb. Hemost.* **1990**, *16 Suppl*, 25–33.
41. Wurm, G.; Tomancok, B.; Nussbaumer, K.; Adelwöhrer, C.; Holl, K. Reduction of ischemic sequelae following spontaneous subarachnoid hemorrhage: A double-blind, randomized comparison of enoxaparin versus placebo. *Clin. Neurol. Neurosurg.* **2004**, *106*, 97–103.
